# Design and Electromagnetic Performance Optimization of a MEMS Miniature Outer-Rotor Permanent Magnet Motor

**DOI:** 10.3390/mi16070815

**Published:** 2025-07-16

**Authors:** Kaibo Lei, Haiwang Li, Shijia Li, Tiantong Xu

**Affiliations:** 1 National Key Laboratory of Science and Technology on Aero-Engine Aero-Thermodynamics, Beihang University, Beijing 100191, China; 201123@buaa.edu.cn (K.L.); 09620@buaa.edu.cn (H.L.); 20374331@buaa.edu.cn (S.L.); 2 Research Institute of Aero-Engine, Beihang University, Beijing 100076, China

**Keywords:** MEMS motor, outer-rotor permanent magnet motor, electromagnetic performance optimization, back EMF, torque, cogging torque, air gap flux density

## Abstract

In this study, we present the design and electromagnetic performance optimization of a micro-electromechanical system (MEMS) miniature outer-rotor permanent magnet motor. With increased attention towards higher torque density and lower torque pulsations in MEMS micromotor designs, an adaptation of an external rotor can be highly attractive. However, with the design complexity involved in such high-performance MEMS outer-rotor motor designs, the ultra-miniature 3D coil structures and the thin-film topology surrounding the air gap have been one of the main challenges. In this study, an ultra-thin outer-rotor motor with 3D MEMS silicon-based coils and a MEMS-compatible manufacturing method for the 3D coils is presented. Additionally, finite element simulations are conducted for the thin-film topology around the air gap to optimize performance characteristics such as torque developed, torque pulsations, and back electromotive force amplitude. Ultimately, the average magnetic flux density increased by 37.1%, from 0.361 T to 0.495 T. The root mean square (RMS) value of the back EMF per phase rises by 14.4%. Notably, the average torque is improved by 11.3%, while the torque ripple is significantly reduced from 1.281 mNm to 0.74 mNm, corresponding to a reduction of 49.9% in torque ripple percentage.

## 1. Introduction

The field of micro-electromechanical systems (MEMS) has witnessed remarkable advancements in recent decades, driven by the increasing demand for miniaturized, high-performance devices across various industries [1]. MEMS technology integrates microelectronics with micro-mechanical components, enabling the development of intricate devices with dimensions ranging from micrometers to millimeters [2,3,4]. Among these MEMS devices, miniature motors have garnered significant attention due to their potential applications in aerospace [5], biomedical [6], automotive, and consumer electronics sectors [7,8]. These applications often require precise motion control, high power density, and compact designs, making the optimization of MEMS motors a critical area of research.

MEMS motors can be classified based on their driving principles, including electrostatic [9,10], piezoelectric [11], and electromagnetic types [12,13,14]. Each type offers distinct advantages and challenges. Electrostatic motors are known for their high precision and low power consumption, but are limited by low torque output and high voltage requirements [15,16]. Piezoelectric motors provide high torque density and fast response times but suffer from relatively low power density and complex driving circuits [17,18]. Electromagnetic motors, on the other hand, offer a promising solution with their high power density and efficiency, making them suitable for applications requiring significant torque output and compact designs [19].

Despite the advantages of MEMS motors, they still face several challenges. While electromagnetic micro motors offer higher power density compared to other motor types, this advantage is insufficient to meet the power and torque requirements of current applications. Currently, most MEMS motors use planar coils, which limit their power density and magnetic flux utilization efficiency [20,21]. Therefore, it is extremely important to develop three-dimensional structure coils using the MEMS process [22].

In addition to manufacturing challenges, the complex topology of MEMS motors also poses significant design difficulties [23,24]. Nevertheless, with advancements in mathematical modeling and computer simulation, numerous papers have studied the optimization of motors. Algorithms such as response surface methodology (RSM) [25,26,27], differential evolution (DE) [28,29], genetic algorithm (GA) [30,31,32], and particle swarm optimization (PSO) [33,34] have been employed in motor optimization. Jo et al. [26] successfully reduced the cogging torque of a BLDC motor using RSM. Moghaddam et al. [27] applied RSM to optimize the stator and rotor parameters of a synchronous reluctance motor, thereby enhancing its performance. Yetgin et al. [28] optimized the stator and rotor slot parameters using DE, achieving minimal stator/rotor inductance and leakage reactance. Bonthu et al. [29] applied DE to determine the optimal shape of the magnetic flux barrier in the stator of an outer rotor permanent magnet synchronous reluctance motor. Nakata et al. [30,31] used GA for motor optimization, coupled with a coarse network to enhance efficiency. Zhou et al. [32] developed an optimization method combining GA that reduced torque ripple by 3.25%. Wang et al. [33] used PSO to reduce electromagnetic vibration in a 6-pole, 9-slot permanent magnet synchronous motor. Gao et al. [34] applied PSO to lower torque ripple in switched reluctance motors by 9.2%.

These optimization initiatives can secure a global optimal solution via a substantial volume of computations, leveraging advanced optimization algorithms and methods. However, the computational time required is substantial. The majority of optimizations zero in on aspects like the shape of permanent magnets (PMs), rotor parameters, and electromotive force harmonics. However, these optimization methods often lack pertinence, with optimization objects and objectives having only a tenuous relationship. In this paper, the geometry around the air gap of the ultra-thin motor is optimized. The correlation between each part and the optimization goal is analyzed, providing a targeted reference for similar motor designs.

This paper is organized as follows. In Section 3, a model of the ultra-thin MEMS outer-rotor motor is proposed, and an ultra-miniature 3D coil structure is designed based on MEMS-compatible fabrication methods. In Section 3, the topology around the air gap of the ultra-thin motor is optimized, and the correlation between all parameters and the optimization objectives is proposed. Section 4 presents the performance of the motor before and after optimization by using the FEM model. It also verifies the effectiveness of optimizing parameters within the relevant ranges.

## 2. Design

### 2.1. Model Design

**Figure 1:**
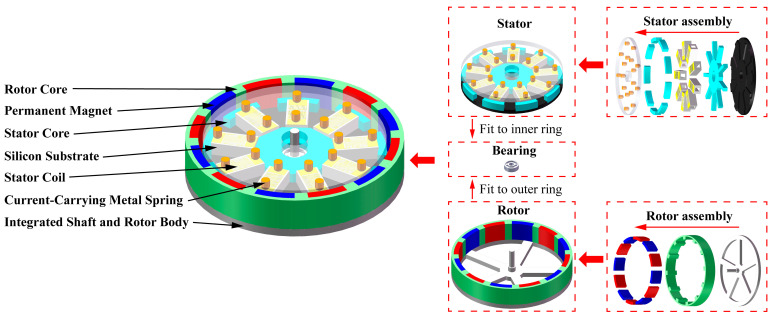
Model and assembly process of the outer-rotor permanent magnet rotary micromotor.

This paper designs an ultrathin three-dimensional (3D) outer-rotor permanent magnet rotary micro-motor model using 3D MEMS silicon-based coils. As shown in Figure 1, the stator comprises a 3D solenoid coil, a load-bearing silicon substrate, an inserted stator irregular iron core, a metal spring for pressing and electrical connection, and an ultrathin hardboard. The rotor consists of a permanent magnet, a rotor core, and an integrated shaft-connected rotor body. When designing these components, it is essential to consider compatibility with MEMS technology. For example, when designing the inserted stator irregular iron core, deep etching in the MEMS coil process can lead to slot expansion and edge deposition, resulting in micro-scale dimensional errors. Therefore, the stator’s irregular iron core design must incorporate micro-scale dimensional redundancy to ensure proper fit and reflect design precision and MEMS compatibility. Similarly, the load-bearing silicon substrate, metal spring, and ultrathin hardboard require micro-scale redundancy design to achieve the structure of the MEMS ultrathin 3D outer-rotor permanent magnet rotary micro-motor.

### 2.2. Pole Number Design

**Figure 2:**
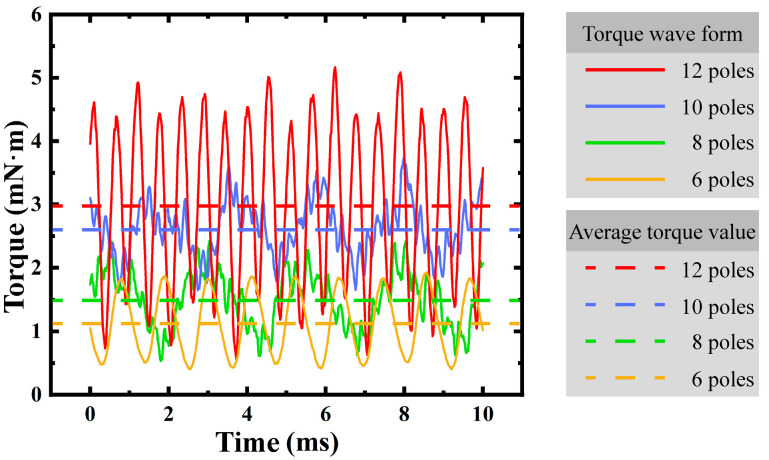
Waveform of torque versus time for different numbers of magnetic poles.

**Figure 3:**
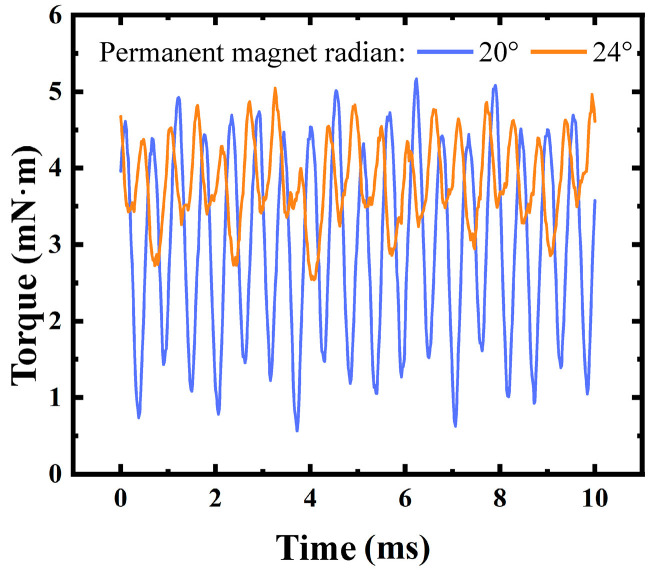
The torque fluctuations can be reduced by changing the pole arc.

In the design of micro motors, the selection of slots and poles is a key factor affecting motor performance. This paper focuses on micro motors compatible with MEMS technology and selects a slot-pole combination of 9 slots and 12 poles. This choice considers the theoretical characteristics of fractional-slot windings, finite element simulation validation, and process limitations. With a fractional-slot ratio of Z/p = 9/12 = 3/4, this combination meets the requirements of fractional-slot concentrated winding, reduces cogging torque, and optimizes slot fill factor and air gap flux density utilization. Finite element simulations (Figure 2) show that the 12-pole structure offers the best air gap flux density utilization and average output torque while keeping the permanent magnet volume constant. Although the 20° pole arc of the 12-pole structure causes harmonic coupling with the stator yoke angle, leading to torque waveform fluctuations, increasing the pole arc coefficient to 24° significantly reduces cogging torque and maintains maximum average torque output, as shown in Figure 3. Moreover, the 9-slot and 12-pole design balances slot width and pole number, satisfying slot fill factor optimization and avoiding process/assembly issues like narrow slots causing dicing difficulties and excessive poles leading to magnetic field saturation. Integrating theoretical analysis, simulation validation, and process limitations, the 9-slot and 12-pole combination is optimal for micro motor design and provides crucial design evidence for multi-objective parameter optimization of MEMS motors.

### 2.3. Fundamental Parameters

Since internationally recognized nano-drones have wingspans between 25 mm and 150 mm [35], drive motors must be under 25 mm in size. In this study, the armature diameter was set at 20 mm for the micro-motor design. As per the model in Figure 1, the stator MEMS coil design aimed to maximize the number of slots and armature reaction while ensuring MEMS-manufacturing compatibility.

**Figure 4:**
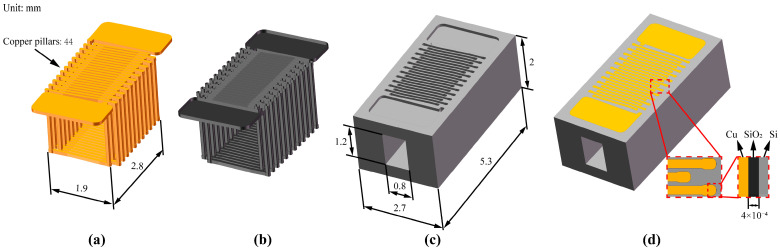
MEMS 3D structure coil model: (**a**) model of electroplated copper coil; (**b**) model of silicon dioxide dielectric layer; (**c**) model of silicon substrate after deep etching and bonding; and (**d**) assembly model along with a detailed close-up.

The MEMS planar coil was transformed into a 3D structure by deep etching holes and slots in the silicon substrate and electroplating copper coils, as shown in Figure 4. To incorporate iron cores, holes were etched in silicon wafers, which were then bonded in pairs. Two 1 mm wafers were used to make 3D coils via MEMS processes, achieving a motor length of just 2 mm and a post-assembly axial length of under 5 mm.

For through-hole fabrication, deep-etching technology was applied. Based on the laboratory’s prior MEMS work, a 10:1 aspect ratio was achievable. A double–sided etching process was designed, each 1 mm wafer requiring a single-sided etch of just 0.5 mm. The through-hole diameter had to exceed 0.05 mm. When designing the coils, the number of turns could not be increased without limit. Considering the armature diameter and leaving a minimum shaft diameter of 1 mm, the maximum number of turns per coil was 44. Based on single-wafer dimensions and circumferential symmetry, the maximum number of slots was 9. With the pole number set at 12 (see Figure 2 and Figure 3), the motor’s fundamental parameters are listed in Table 1.

### 2.4. Manufacturing Method Design

**Figure 5:**
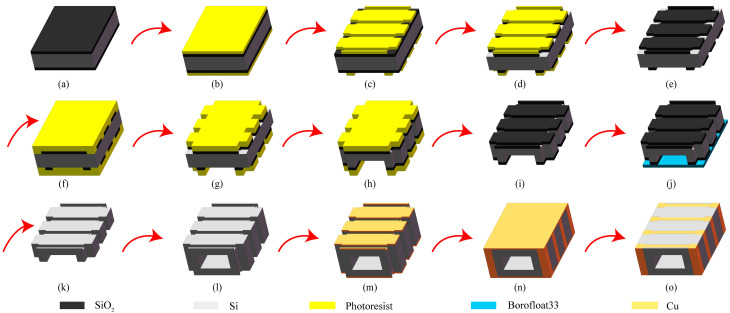
Processing step diagrams of the ultra-miniature 3D coils: (**a**) oxidized silicon wafer preparation; (**b**) thin photoresist coating; (**c**) double-sided pattern exposure; (**d**) double-sided oxide etching by BOE; (**e**) thin photoresist removal; (**f**) thick photoresist coating; (**g**) double-sided alignment exposure; (**h**) double-sided silicon etching; (**i**) thick photoresist removal; (**j**) substrate bonding, and upper etching; (**k**) oxide layer removal by HF; (**l**) silicon-silicon bonding; (**m**) double-sided magnetron sputtering; (**n**) electroplating; (**o**) grinding.

The main challenge in the fabrication method design lies in the ultra-miniature 3D coils. This subsection focuses on the fabrication of coils compatible with MEMS processes. The coils embedded in silicon are relatively complex, but almost all of them are composed of a combination of coil grooves and coil holes. Therefore, Figure 5 uses 3D modeling technology to introduce the process design flow. Only simplified coil grooves and coil holes are shown in the figure to illustrate the detailed parts of the process. The specific processing step diagrams and explanations are as follows:

(a) Prepare two pieces of intrinsic silicon wafers with double-side oxidation, ensuring that they meet the process requirements, with a thickness of 1 mm and an oxide layer thickness of 2 μm.
(b) Apply a thin photoresist on both sides of the silicon wafer. First, perform pre-baking, and then apply the photoresist. The spin-coating speed and time parameters are 4000 rpm for 50 s. After that, put it into an oven and bake at 115 °C for 30 min to dry it for subsequent exposure.
(c) Expose the pattern on both sides once. First, cool the silicon wafer to room temperature, and expose different patterns on the upper and lower surfaces, respectively, with an exposure time of 3 s, and then perform the development operation.
(d) Double-sided BOE (Buffered Oxide Etch). Soak the exposed silicon wafer in a BOE solution (mainly composed of components such as hydrofluoric acid (HF) and ammonium fluoride (NH_4_F)). Remove the exposed oxide layer. Observe under a microscope. When colored stripes appear, try to remove them until the oxide layer is completely removed.
(e) Remove the thin photoresist on both sides. Use a piranha solution (concentrated sulfuric acid (H_2_SO_4_) and hydrogen peroxide (H_2_O_2_) in a ratio of 3:1) to wash off the photoresist, then rinse it thoroughly with deionized water, and finally dry it with a spin dryer.
(f) Apply a thick photoresist on both sides of the silicon wafer. The spin-coating speed and time parameters are 3000 rpm for 50 s. Control the flow rate and position of the photoresist during spin-coating to ensure a uniform photoresist layer. After that, put it into an oven and bake at 95 °C for 60 min.
(g) Double-sided alignment exposure of the secondary pattern. First, cool the silicon wafer to room temperature, and expose different patterns on the upper and lower surfaces, respectively, with an exposure time of 13 s. Ensure accurate alignment of the patterns, and then perform the development operation.
(h) Double-sided etching. First, perform shallow etching of the alignment marks, and then perform deep etching after covering. Control the time and conduct intermittent depth measurements. Adjust the etching parameters according to the situation during the etching process to ensure the etching depth and shape. Complete the etching of the coil holes and the deep grooves for inserting the iron core.
(i) Remove the thick photoresist on both sides, ensuring that there is no residue on the surface of the silicon wafer.
(j) Attach the substrate to the lower surface and etch the upper surface, with an etching depth of 100 μm, to complete the etching of the coil grooves.
(k) Clean the silicon wafer with HF to remove the oxide layer. Use an HF solution to clean the silicon wafer and remove the surface oxide layer, and then rinse it thoroughly with deionized water to ensure that the oxide layer is completely removed, so as to prepare for the subsequent process.
(l) Double-layer silicon-silicon bonding, ensuring tight bonding without bubbles and voids, to form an integral structure.
(m) Double-sided magnetron sputtering. Perform magnetron sputtering operations on both sides of the silicon wafer and sequentially deposit metallic titanium and metallic copper. Control the sputtering parameters to ensure uniform thin-film thickness and no warping in subsequent tests.
(n) Electroplating. Perform an electroplating operation on the surface of the silicon wafer to deposit the required electroplating layer. Ensure that the electroplating layer has a uniform thickness, good adhesion, and is dense without holes.
(o) Grinding. Perform a grinding operation on the silicon wafer to remove the excess electroplated metal, so that each turn of the coil is separated from the other. At the same time, ensure the flatness of the surface, facilitating subsequent operations such as inputting current. So far, all the MEMS processes for the ultra-miniature 3D coils embedded in silicon are completed.

### 2.5. Magnetic Density Design

**Figure 6:**
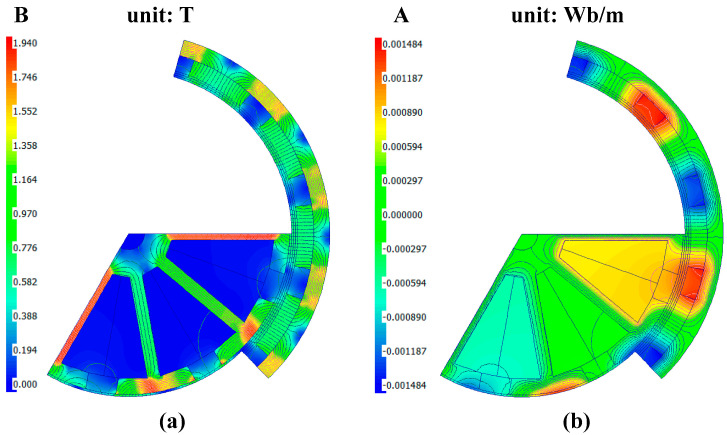
The magnetic flux density of the MEMS micromotor: (**a**) magnetic density distribution; (**b**) vector magnetic potential distribution.

Based on the preliminary fundamental parameters, the magnetic flux density of the micro-motor was solved, and the results are illustrated in Figure 6. The magnetic flux density (B) distribution is depicted in Figure 6a, spanning a range from 0.000 to 1.940 T in 0.194 T increments. The B field follows a radial distribution pattern, indicative of the motor’s electromagnetic topology, with magnetic flux lines circulating from the PM through the air gap to form closed loops with the stator teeth. The region near the PM and stator teeth exhibits the highest intensity, with the PM area registering the maximum B value of up to 1.94 T, reflecting a strong radial magnetic flux. The concentration of magnetic flux lines around the PM and stator teeth confirms the effective magnetic flux guidance by the stator core. In contrast, areas with lower magnetic permeability, such as the air gap and stator slots, display reduced B values, which partially clarifies the origin of the motor’s cogging torque. Figure 6b presents the magnetic vector potential (A) distribution, which is also radial and ranges from −0.001484 to 0.001484 Wb/m in 0.000297 Wb/m increments. The A field intensity peaks near the PM and stator teeth, reaching up to 0.001484 Wb/m in the PM region. The A field mirrors the magnetic flux potential, with higher values corresponding to greater magnetic flux. The magnetic flux lines are concentrated in the PM and stator teeth regions, further confirming the effective magnetic flux guidance by the stator core and forming closed loops from the PM through the air gap to the stator teeth. These visualizations offer a comprehensive understanding of the motor’s magnetic field characteristics.

## 3. Method

This study examines numerous topological structures of micro-motors. Due to their small size, there are critical values in electromagnetic design where topological structure optimization patterns are unclear. We focused on analyzing the impact of topological structure. The temperature of 70.3 °C was used as the motor’s operating temperature during electromagnetic simulations. There are some modeling boundary conditions.

- The external boundary was set to an air domain that fully encapsulates the motor.
- The rotor was defined as the rotating component with an initial phase angle of 0°.
- The rotational speed was set to the standard speed of 3000 rpm.
- The excitation source was defined as a sinusoidal current drive with an effective value of 0.5 A.
- The winding connection method was set to a star connection.
- The load type was assumed to be a constant torque load.
- Assembly errors were not considered, and a uniform air gap was assumed.
- The material properties of the stator and rotor (such as silicon steel sheets of M350–50A and permanent magnets of N30UH) were set to the standard parameters in the software’s built-in material library.

**Figure 7:**
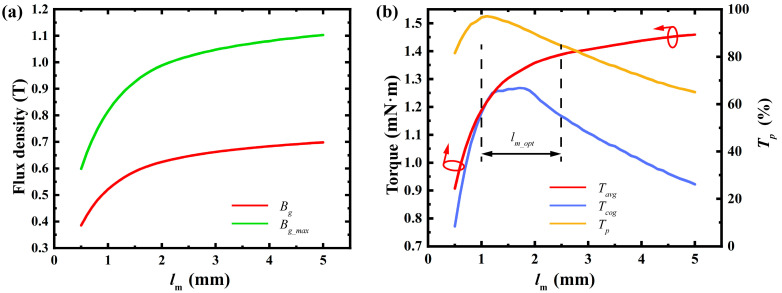
Effect of radial thickness of permanent magnet: (**a**) variation in maximum air gap flux density and average air gap flux density; (**b**) variation in average torque, cogging torque, and torque ripple percentage. (Arrows indicate the optimization trend of the corresponding parameter).

**Figure 8:**
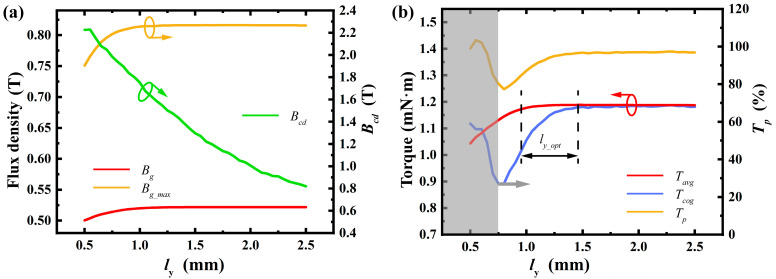
Effect of stator slot shoulder height: (**a**) variation in maximum air gap flux density, average air gap flux density, and tooth top flux density; (**b**) variation in average torque, cogging torque, and torque ripple percentage. (Arrows indicate the optimization trend of the corresponding parameter).

**Figure 9:**
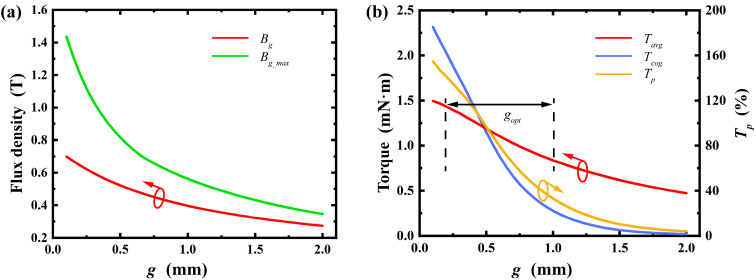
Effect of air gap length: (**a**) variation in maximum air gap flux density and average air gap flux density; (**b**) variation in average torque, cogging torque, and torque ripple percentage. (Arrows indicate the optimization trend of the corresponding parameter).

**Figure 10:**
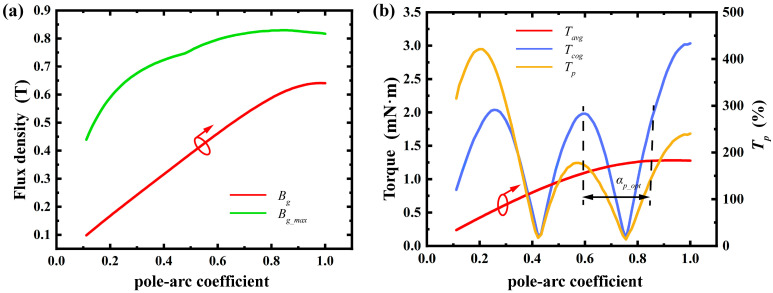
Effect of arc length of permanent magnets: (**a**) variation in maximum air gap flux density and average air gap flux density; (**b**) variation in average torque, cogging torque, and torque ripple percentage. (Arrows indicate the optimization trend of the corresponding parameter).

**Figure 11:**
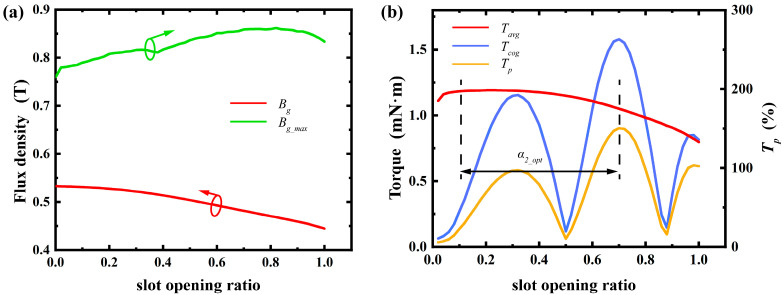
Effect of slot opening arc length: (**a**) variation in maximum air gap flux density and average air gap flux density; (**b**) variation in average torque, cogging torque, and torque ripple percentage. (Arrows indicate the optimization trend of the corresponding parameter).

**Figure 12:**
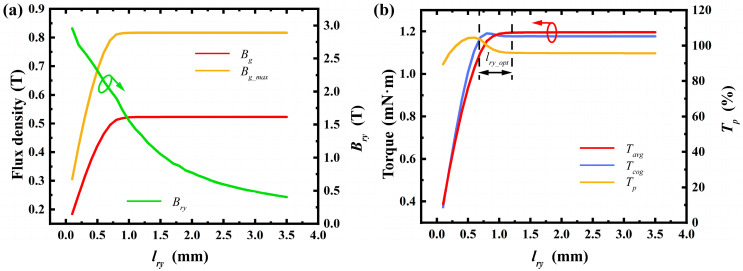
Effect of thickness of rotor core hub: (**a**) variation in maximum air gap flux density, average air gap flux density, and rotor yoke flux density; (**b**) variation in average torque, cogging torque, and torque ripple percentage. (Arrows indicate the optimization trend of the corresponding parameter).

By methodically analyzing and adjusting topological structures such as the radial thickness of permanent magnets, stator slot shoulder height, air gap length, and material selection, we laid a solid groundwork for enhancing motor performance. This analysis clarifies each topological structure’s impact range and allows for optimization based on specific trends. For instance, optimizing the permanent magnet thickness boosts average torque while cutting material waste. Adjusting the slot opening arc length and rotor pole arc coefficient reduces cogging torque, improving motor stability. Below is a detailed discussion on the influence of topological structure design. In the Figure 7, Figure 8, Figure 9, Figure 10, Figure 11 and Figure 12, the ‘_opt’ denotes the adjustment range. The main electromagnetic performance goals for micro-motors are average torque, torque ripple, and cogging torque. Table 2 lists the key symbols.

1. Radial Thickness of Permanent Magnet:

As the radial thickness of the permanent magnet increases, both the average and maximum air gap flux densities rise rapidly at first and then gradually level off. This indicates that when the permanent magnet is thin, increases in thickness significantly affect the air gap flux density and average torque. However, beyond a certain thickness, these effects diminish. To balance performance and material cost, the optimal thickness range is identified as 20% to 50% of the torque rise rate. Within this range, variations in permanent magnet thickness have minimal impact on cogging torque and torque ripple, ensuring smooth motor operation. Figure 7 illustrates the close relationship between permanent magnet thickness and the trends in air gap flux density and average torque.

2. Stator Slot Shoulder Height:

Variations in the stator slot shoulder height have a relatively small impact on the average air gap flux density but significantly affect the maximum air gap flux density and the waveform of the air gap flux density distribution. If the stator slot shoulder is too thin, it may cause distortion in the air gap flux distribution, thereby affecting the motor’s performance. Additionally, an overly low stator slot shoulder height might lead to excessively high magnetic flux density in the stator core, approaching or exceeding the saturation level of silicon steel sheets, which can result in motor performance degradation. Therefore, it is crucial to ensure sufficient stator slot shoulder height during the design phase. This prevents magnetic saturation and optimizes the air gap flux distribution waveform, enhancing the motor’s overall performance. As shown in Figure 8, an ideal air gap flux distribution is achieved when the stator slot shoulder height is above 1 mm.

3. Air Gap Length:

The air gap length significantly impacts motor performance. As it increases, the average torque decreases due to reduced magnetic coupling between the permanent magnet and stator, while torque ripple also decreases, enhancing motor smoothness. In design, an optimal air gap length must be chosen by balancing average torque and torque ripple, while considering manufacturing feasibility. Figure 9 shows the notable effects of air gap length variations on these parameters.

4. Arc Length of Permanent Magnet:

Changes in the arc length of permanent magnets are reflected through the pole arc coefficient. Increasing this coefficient raises both the average air gap flux density and torque output. However, it can also boost cogging torque and torque ripple, jeopardizing motor stability. So, when optimizing the permanent magnet’s arc length, it is crucial to balance average torque and torque ripple for the best pole arc coefficient, as shown in Figure 10.

5. Slot Opening Arc Length:

Slot opening arc length variations significantly affect cogging torque and torque ripple, with a smaller impact on average output torque. A smaller slot opening arc length increases average air gap flux density but reduces its variation near the slot edges, lowering maximum air gap flux density. As the slot opening arc length increases, the tooth’s magnetic conduction area decreases. The edge effect causes the magnetic path to favor the tooth core, raising tooth magnetic flux density and impacting maximum air gap flux density. Therefore, when optimizing the slot opening arc length, focus on its impact on torque ripple to ensure smooth motor operation. As shown in Figure 11, the slot opening ratio below 0.7 has minimal effect on average torque but substantially influences torque ripple.

6. Thickness of Rotor Core Hub:

The rotor core yoke thickness significantly impacts the air gap flux density and torque performance. As the yoke thickness increases from 0, the air gap flux density rises until a certain value is reached, beyond which it plateaus. This indicates that increasing the yoke thickness can enhance the air gap flux density and torque performance when the yoke is relatively thin, but further increases beyond a certain thickness yield minimal performance benefits. Therefore, optimizing the rotor core yoke thickness requires selecting an appropriate range to avoid material waste while maximizing performance. As shown in Figure 12, the air gap flux density saturates around a yoke thickness of 0.8 mm, beyond which further increases offer limited performance improvements.

## 4. Results

Based on the relationships between parameters and optimization objectives obtained in the previous section, the topology was optimized within the relevant range. This led to significant improvements in the electromagnetic performance indicators of the MEMS micromotor, as presented below.

**Figure 13:**
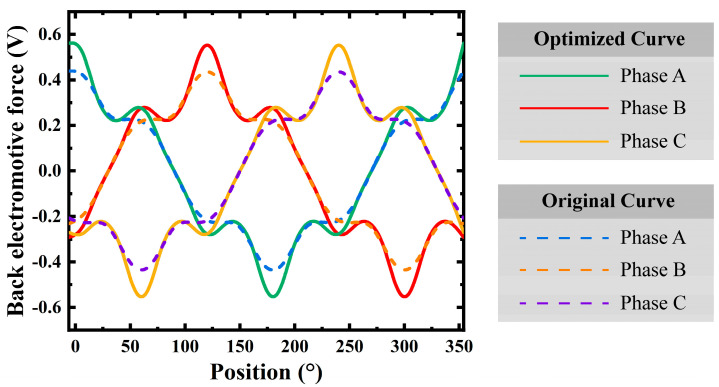
Optimization of back electromotive force performance.

The optimization process has improved the back electromotive force (EMF) characteristics of the micro motor. As shown in Figure 13, the optimized curves (solid lines) for phases A, B, and C, when compared to the original curves (dotted lines), exhibit higher back EMF amplitudes at a 360° electrical angle, indicating consistent performance enhancement.

**Figure 14:**
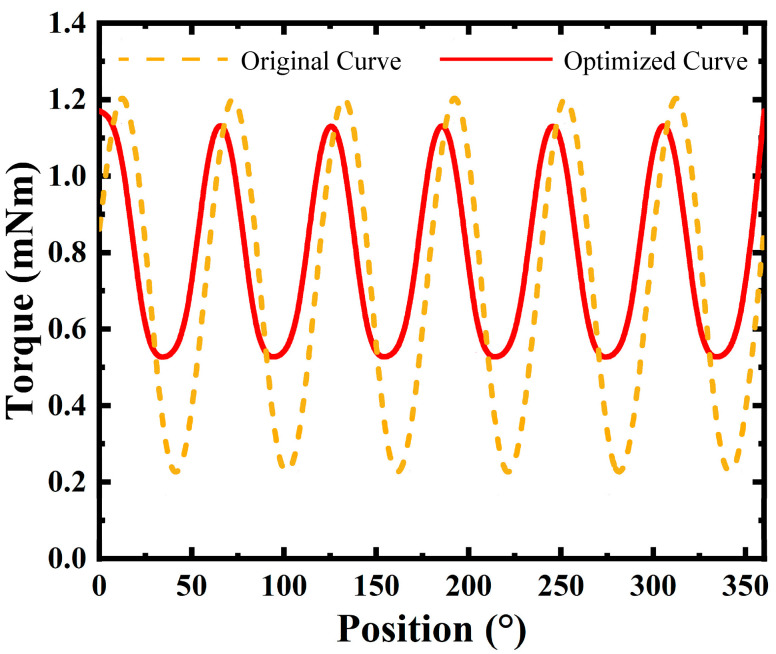
Optimization of torque performance.

Figure 14 illustrates the torque performance before and after optimization. The optimized curve (solid red line) demonstrates a more uniform torque distribution across the position range. The average torque values are slightly increased, while the fluctuations are significantly reduced. This enhanced torque profile contributes to better mechanical performance and reduced pulsation, which is crucial for applications requiring precise motion control.

**Figure 15:**
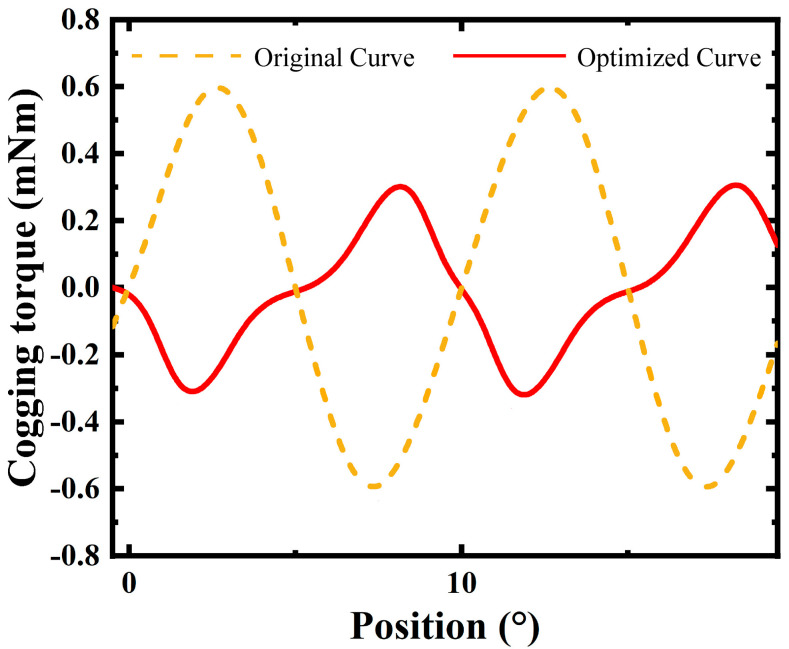
Optimization of cogging torque performance.

The cogging torque, which is a significant factor affecting the smooth operation of electrical machines, has been substantially reduced through optimization. Figure 15 shows a comparison of the cogging torque curves. The optimized curve (solid red line) exhibits lower amplitude variations and reduced peak values compared to the original curve (dashed yellow line). This reduction in cogging torque leads to smoother rotation and minimized vibration and noise during operation.

**Figure 16:**
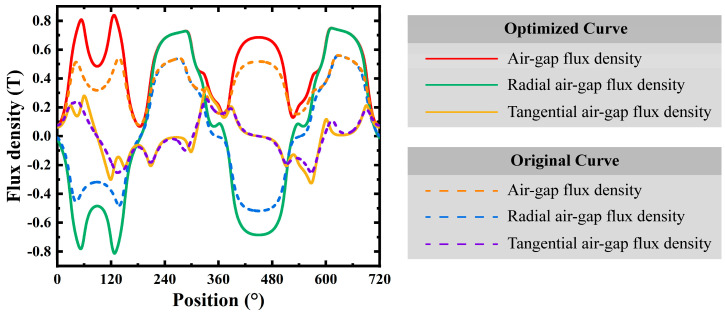
Optimization of air gap magnetic flux density performance.

The air gap magnetic flux density is a critical parameter influencing the machine’s performance. Figure 16 presents the flux density distributions for the original (dashed lines) and optimized (solid lines) curves. The optimized curves show an enhanced magnetic field distribution. In particular, the radial component of the flux density has been significantly strengthened, resulting in reduced leakage effects.

**Figure 17:**
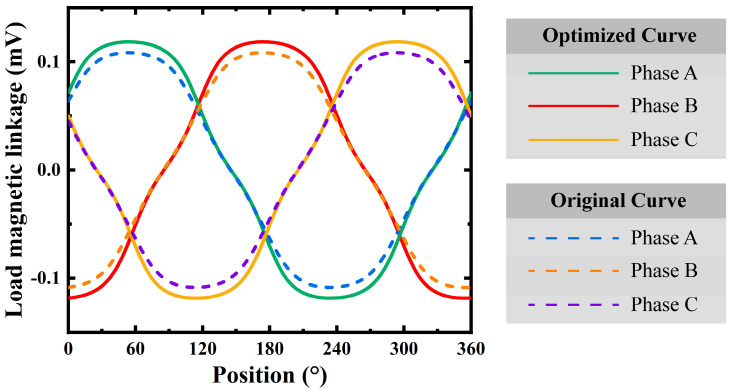
Optimization of load magnetic linkage performance.

The load magnetic linkage, which affects the energy transfer efficiency, has also been optimized. Figure 17 displays the load linkage curves for the three phases. The optimized curves (solid lines) exhibit a more sinusoidal waveform with reduced distortion. This improvement enhances the machine’s ability to handle loads efficiently, resulting in better power delivery and reduced losses.

These results highlight the effectiveness of the topology optimization strategy, which has significantly enhanced the electromagnetic performance of the micro motor. The average magnetic flux density increased from 0.361 T to 0.495 T, a 37.1% improvement. The RMS value of the back EMF per phase rose from 0.153 V to 0.175 V, an increase of 14.4%. The average torque was boosted from 0.708 mNm to 0.788 mNm, up by 11.3%. Moreover, the cogging torque ripple was significantly reduced from 1.281 mNm to 0.74 mNm, which led to a significant reduction in torque ripple percentage, with a decrease of 49.9%.

## 5. Conclusions

This study presents an ultra-thin outer-rotor motor with 3D MEMS silicon-based coils and a MEMS-compatible manufacturing method for the 3D coils. Additionally, this study conducts finite element simulations for the thin-film topology around the air gap to optimize performance characteristics such as developed torque, torque pulsations, and back electromotive force amplitude. The average magnetic flux density, a key determinant of motor efficiency, increased significantly by 37.1%. The RMS value of the back EMF per phase rose by 14.4%, enhancing the motor’s power generation capability. Mechanical performance was bolstered by an 11.3% increase in average torque. Most strikingly, the torque ripple percentage, which directly impacts the smoothness of operation, was reduced by 49.9%. The optimized MEMS micromotor demonstrates superior performance in terms of efficiency, torque production, and operational smoothness, making it highly suitable for advanced applications such as nano-drones and precision instruments.

## Figures and Tables

**Table 1:** Basic micromotor parameters.

Parameter	Value
Number of Pole Pairs	6
Number of Slots	9
Armature Diameter	20 mm
Shaft Radius	0.5 mm
Motor Length	2 mm
Copper Pillars of a Silicon Substrate Coil	44
Input Current Waveform	Sine Wave
Input Current Amplitude	0.5 A

**Table 2:** Symbols.

Symbol	Meaning
*l*_m_	Radial thickness of permanent magnet
*h*_m_	Height of the permanent magnet
*l*_y_	Stator slot shoulder height
*g*	Air gap length
*B*_g_max_	Maximum air gap flux density
*B*_g_	Average air gap flux density
*B*_cd_	Tooth top flux density
*B*_ry_	Rotor yoke flux density
*α*_1_	Arc length of the permanent magnet
*α*_2_	Slot opening arc length
*l*_ry_	Thickness of rotor core hub
*T*_avg_	Average torque
*T*_cog_	Cogging torque
*T*_p_	Torque ripple percentage

## Data Availability

The original contributions presented in this study are included in the article. Further inquiries can be directed to the corresponding author.
